# A Big Coal Block Alarm Detection Method for Scraper Conveyor Based on YOLO-BS

**DOI:** 10.3390/s22239052

**Published:** 2022-11-22

**Authors:** Yuan Wang, Wei Guo, Shuanfeng Zhao, Buqing Xue, Wugang Zhang, Zhizhong Xing

**Affiliations:** 1 College of Mechanical Engineering, Xi’an University of Science and Technology, Xi’an 710054, China; 2 School of Cyber Engineering, Xidian University, Xi’an 710071, China

**Keywords:** scraper conveyor, coal block congestion, big size, YOLO-BS, transform

## Abstract

With the aim of solving the problem of coal congestion caused by big coal blocks in underground mine scraper conveyors, in this paper we proposed the use of a YOLO-BS (YOLO-Big Size) algorithm to detect the abnormal phenomenon of coal blocks on scraper conveyors. Given the scale of the big coal block targets, the YOLO-BS algorithm replaces the last layer of the YOLOv4 algorithm feature extraction backbone network with the transform module. The YOLO-BS algorithm also deletes the redundant branch which detects small targets in the PAnet module, which reduces the overall number of parameters in the YOLO-BS algorithm. As the up-sampling and down-sampling operations in the PAnet module of the YOLO algorithm can easily cause feature loss, YOLO-BS improves the problem of feature loss and enhances the convergence performance of the model by adding the SimAM space and channel attention mechanism. In addition, to solve the problem of sample imbalance in big coal block data, in this paper, it was shown that the YOLO-BS algorithm selects focal loss as the loss function. In view of the problem that the same lump coal in different locations on the scraper conveyor led to different congestion rates, we conducted research and proposed a formula to calculate the congestion rate. Finally, we collected 12,000 image datasets of coal blocks on the underground scraper conveyor in Daliuta Coal Mine, China, and verified the performance of the method proposed in this paper. The results show that the processing speed of the proposed method can reach 80 fps, and the correct alarm rate can reach 93%. This method meets the real-time and accuracy requirements for the detection of abnormal phenomena in scraper conveyors.

## 1. Introduction

**Figure 1:**
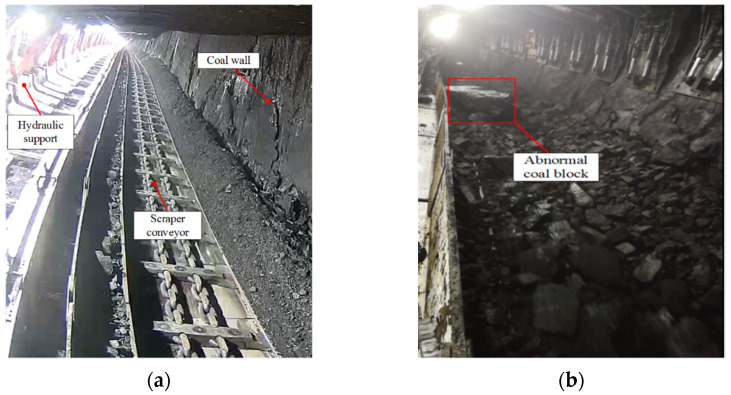
Scraper conveyor: (**a**) underground scraper conveyor; (**b**) unusual coal lumps in coal stream.

Mining scraper conveyors are used to transport initial coal bodies which have been newly mined using shearers, as shown in Figure 1. Scraper conveyors are widely used in the field of coal mine material transportation because they have long conveying distances and large conveying capacities [1,2,3,4]. Shearers have high working efficiency when mining big coal blocks and low efficiency when mining small coal blocks. The mining efficiency and mining intensity vary, as a shearer will mine unevenly sized coal during the working process. However, big coal blocks in a mined coal body will cause the coal outlet of the scraper conveyor to be blocked, resulting in excessive coal accumulation in the trough, and then, the coal body will overflow. In addition, the scraper conveyor may cause increased wear and tear to the scraper conveyor chain and equipment when transporting big coal blocks. With the development of intelligent mining-related technologies, more stringent requirements for coal mining efficiency have been proposed, meaning the real-time monitoring of big coal blocks in scraper conveyors must be conducted urgently.

With the aim of enabling big coal blocks to be monitored in scraper conveyors, experts have mainly studied this issue from two perspectives: the monitoring of coal flow in scraper conveyors and the monitoring of big coal blocks in scraper conveyors. Most of the initial coal flow detection methods are based on contact sensors such as electronic tape scales and nuclear scales.

These devices include weighing frames, load cells, speed sensors, and totalizer meters. The advantages of this kind of method [5,6,7,8] include its low cost, simple and convenient installation, and the fact that it does not require many modifications to be made to the original equipment base site. However, due to the humidity around the sensor, the sensor is prone to short-circuit, and when the sensor is faulty or the probe electrostatically conducts the circuit, it can be mistakenly identified as a coal pile, which means the flow monitoring accuracy is not high. To overcome the shortcomings of the contact method, scholars have successively proposed coal flow calculation methods based on non-contact scraper conveyors with ultrasonic technology [9] and laser technology [10,11]. The laser scanning method is a new material flow measurement method which can accurately measure coal flow, but it is not popular due to the high cost. The detection side of the rangefinder based on the ultrasonic principle also has poor anti-interference performance and high accuracy.

With the extent of experts’ research into the problem of coal flow detection in scraper conveyors, it has gradually been found that it is impractical to monitor big coal blocks from coal flows. In recent years, scholars have conducted in-depth research regarding big coal block recognition through machine vision technology, which is divided into two main categories: image processing algorithms and deep learning algorithms.

In terms of traditional image processing research, Sun et al. [12] established a digital image analysis method for use on coal rock interfaces based on the textural characteristics of coal rock. Zhang et al. [13] introduced a method of image analysis to predict the ash content of coarse coal by considering the prediction effect of ash content of different particle sizes. These studies all used traditional image processing techniques to extract edges and used thresholds to identify coal lumps and provide early warnings of coal piles, but these techniques have low detection accuracy, poor real-time performance, and an inability to accurately judge coal piles.

The deep learning algorithm has a high recognition rate and strong robustness, and has been rapidly disseminated in coal block identification. Wang et al. [14] proposed the video detection of foreign objects on a belt surface based on SSD to solve the problem of damage to the belt conveyor due to foreign objects in coal mine belt conveyors. Zhang et al. [15] introduced the attention mechanism in a decoder to capture rich semantic information.

With the aim to solve the problem that big coal blocks in a scraper conveyor cannot be monitored accurately and the real-time performance cannot meet the requirements, we proposed a big coal block monitoring method based on the YOLO-BS algorithm. In this method, real-time video data of big coal blocks on a scraper conveyor are collected through a camera, and then the YOLO-BS algorithm proposed in this paper is used to identify the big coal blocks and monitor them. The main contributions of this paper are as follows:

(1) The transform module that can extract global information of an image is integrated with the YOLOv4 algorithm, meaning the YOLO-BS algorithm has a strong ability to read global information.
(2) In response to the requirement to monitor big coal blocks in scraper conveyors, the PAnet module of the YOLO-BS algorithm performs the pruning operation of small targets, and the simAM module is introduced to accelerate the convergence of the model and reduce the feature loss.
(3) Sample imbalance was present in the big coal block data collected on site. In this paper, focal loss was selected as the loss function of YOLO-BS to solve the problem of data imbalance.
(4) To verify the effectiveness of the scraper conveyor monitoring algorithm proposed in this paper, we installed data acquisition and processing equipment in Daliuta Mine as an example of its application.

The second section of the article mainly reviews the literature regarding the current methods used to detect big coal blocks. The technical route studied in this paper is presented in the third section. The fourth section of this paper mainly introduces the proposed scraper conveyor detection algorithm, YOLO-BS, which was used to solve the coal monitoring problem, and the method of coal outlier calculation. The specific implementation details regarding big coal blocks monitoring and the example application of the method proposed in this paper are presented in the fifth section. The sixth section presents the inadequacies of this study and summarizes the full text. We have uploaded the code of the YOLO-BS algorithm to GitHub at https://github.com/wy121212/YOLO-BS.git (access on 1 January 2022).

## 2. Literature Review

In this paper, coal size was detected in the working scraper conveyor, and the methods commonly used to achieve this can be divided into two categories: coal block detection methods, which mainly include traditional image processing methods, and image processing methods based on deep learning.

### 2.1. Traditional Image Processing Methods

In the study of traditional image features, Xian et al. [16] improved the target detection accuracy by improving the target detection method of the Gaussian mixture model. Hou et al. [17] proposed a method for the recognition of coal gangue based on a neural network by analyzing the differences in surface textures and grayscale characteristics between coal and gangue. Zhang et al. [18] combined Hough transform, shadow, and Harris corner detection methods to achieve target detection in the subsidence area of subsidence pits and troughs in the regional ground collapse phenomenon caused by mining. To realize the real-time and accurate measurement of coal quantity, Wang et al. [19] proposed a coal quality detection and classification method based on machine vision and deep learning. Bai et al. [20] proposed an image segmentation method for coal particle images. Wang et al. [21] designed a traditional algorithm for the fine segmentation of coal gangue images, which combined the star edge detection method and the morphological method.

### 2.2. Image Processing Method Based on Deep Learning

Considering ubiquitous fine-grained features in industrial object images, Lv et al. [22] proposed a single-shot fine-grained object detector and applied it to coal gangue images in coal preparation plants. Yan [23] used the YOLOv5 algorithm to analyze spectral images of coal gangue. Liu et al. [24] proposed an improved YOLOv4 algorithm that uses a Laplacian operator and Gaussian filter to reduce mine dust and impact, and has good anti-interference ability. Wang et al. [25] used the improved YOLOv3 algorithm to detect obstacles and only output the type and position of obstacles that coincided with the dangerous area. Ma’s team [26] applied the CenterNet object detection algorithm to the detection of foreign objects in coal conveying belts of coal mines. With the aim of achieving a fast-running speed of the coal conveying belt and lessening the influence of background and light sources on detected objects, an improved CenterNet algorithm was proposed. Dong et al. [27] proposed an improved target detection model based on tiny-yolov3, which improved the obstacle detection performance under real-time detection conditions. Qu’s team [28] constructed a conveyor belt damage detection method based on an ADCN (adaptive deep convolutional network). Shao [29] proposed a paper jam detection method at the transfer point based on an improved Mask R-CNN. Xie et al. [30] proposed an image expansion method based on a deep convolutional generative adversarial network (DCGAN). Zhao Xiaohu et al. [31] adopted a principal component analysis network (PCANet) and planar neural network (FNN)-based coal and foreign body recognition method in coal mine conveyor belt images to improve the efficiency of coal mine foreign body detection. Based on the YOLOv3detection algorithm, Gui et al. [32] established a deformable convolution YOLOv3 network model to solve the problem of coal gangue detection and identification by means of deformable convolution, the multi-k-means clustering result averaging method, and data enhancement technology. However, this algorithm has some limitations, such as a limited receptive field, a slow convergence speed, and low recognition accuracy for small particles. Liu et al. [33] proposed a new method of fusing image spatiotemporal features and directly using live surveillance video images for belt damage identification. The method was based on a deep learning network structure, and an end-to-end belt damage detection model was designed. Wei et al. [34] built a network for the automatic detection of forging defects based on YOLO and GAN algorithms.

## 3. Research Technology Route

**Figure 2:**
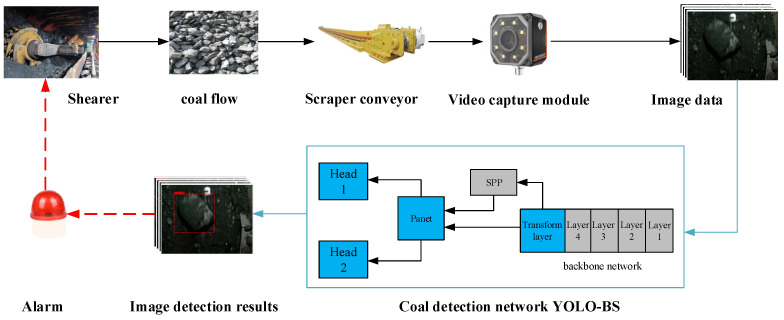
Technical route of coal monitoring research.

Inspired by research in the existing literature, in this paper, we studied the problem of monitoring bulk coal in scraper conveyors. This study can be divided into three sections. Firstly, we obtained information regarding big coal blocks by installing a high-speed camera directly above the scraper conveyor to regularly collect images. Secondly, the YOLO-BS algorithm proposed in this paper was used to detect images of coal blocks. Finally, through the analysis of the coal block detection results, it was determined whether an alarm was needed to complete the feedback control of the shearer. The research process is shown in Figure 2.

## 4. Method

### 4.1. Big Coal Block Detection Algorithm for Scraper Conveyor

#### 4.1.1. YOLO-BS Algorithm Framework

**Figure 3:**
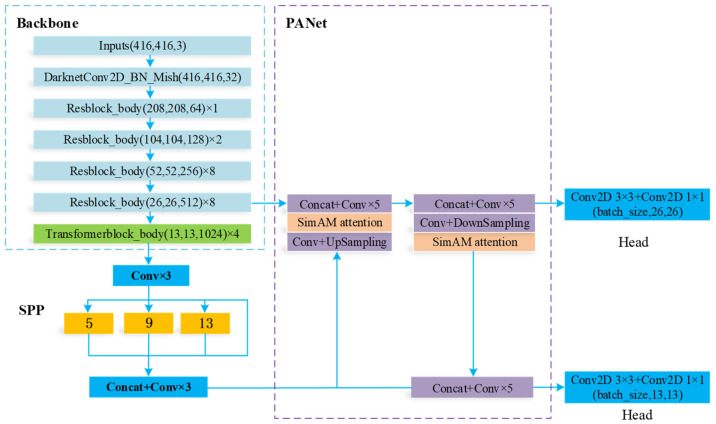
YOLO-BS algorithm structure framework.

A YOLO-BS algorithm was proposed to solve the problem of monitoring big coal blocks. The YOLO-BS algorithm has a backbone network, SPP module [35], PANet module, and two YOLO heads, as shown in Figure 3. A backbone network containing visual transforms is used to extract feature information from the input image. The SPP module can mine deeper information from the backbone network for feature extraction. The PANet network module bidirectionally fuses the features output by the backbone network and the features output by the SPP module, and it outputs them to two YOLO-Heads. Two large-scale YOLO-Heads can identify coal objects of different scales.

#### 4.1.2. The Backbone Structure of Fusion Transform

The YOLO-BS algorithm is composed of a fusion of CSPDarkNet53 and a visual transform module [36]. The size of the image input by the backbone network is normalized to 416 × 416 × 3, and the first layer consists of the DarknetConv2D_BN_Mish convolution module, which does not change the size of the image to change the feature map channel to 32. The second layer consists of a residual module, and the size of the input feature map is 208 × 208 × 64. The third, fourth, and fifth layers correspond to 2, 8, and 8 residual modules, which change the size of the input feature map to 104 × 104 × 128, 52 × 52 × 256, and 26 × 26 × 512, respectively.

When the feature map is input to the transform module, the size of the feature map is 13 × 13 × 1024. Before entering the transform module, the feature map needs to be thin and flattened to 169 × 1024, so that the transform module can learn the global information from the input image.

**Figure 4:**
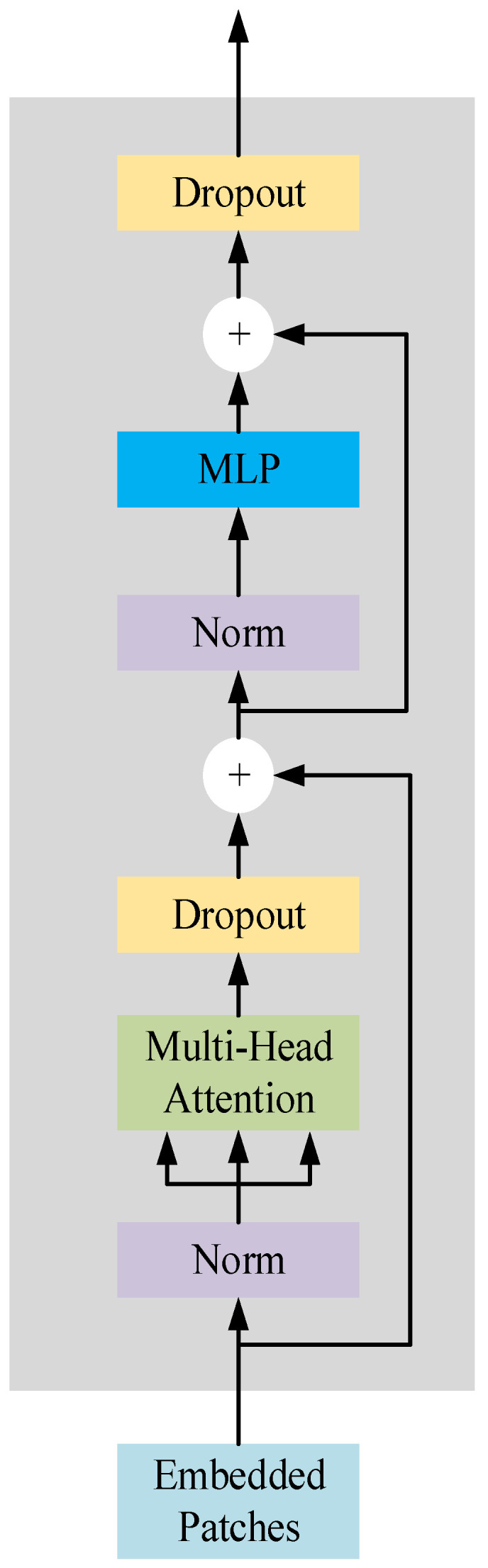
Vision transformer encoder module.

The visual transform module used in this paper consists of four transform encoders, as shown in Figure 4. The transform encoder consists of two residual structures. The first residual structure contains layer norm, Multi-Head Attention, and Drpout modules, and the second residual structure contains three modules: layer norm, MLP [37] block, and Drpout. The layer norm module ensures that the meaning vectors converted from words in each sequence are on the same scale. Multi-Head Attention can map the input to different subspaces through different linear changes, so that the model can understand the input sequence from different perspectives. MLP is a feedforward artificial neural network model that maps multiple input datasets to a single output dataset. The Drpout module is used to improve the overfitting problem of the model.

#### 4.1.3. PANet Modules with Large-Scale Features

The up-sampling and down-sampling operations are performed in the PANet module of the YOLO-BS algorithm, in which feature loss easily occurs. By adding the SimAM [38] space and channel attention mechanism, this problem can be effectively improved, and the convergence performance of the model can be simultaneously enhanced.

The attention mechanism of SimAM improves the attention ability of the model by evaluating the importance of neurons. Normally, neurons with the spatial inhibition effect are more valuable, because this SimAM module provides a method through which important neurons can be found. The energy function is defined as follows:

(1) $$e_{t}\left(w_{t},b_{t},y,x_{i}\right)={\left(y_{t}-\hat{t}\right)}^{2}+\frac{1}{M-1}\sum_{i=1}^{M-1}{\left(y_{o}-{\hat{x}}_{i}\right)}^{2}$$

In Equation (1), $\hat{t}=w_{t}t+b_{t},\hat{x_{i}}=w_{t}x_{i}+b_{t}$. Equation (1) is the linear separability between neurons in the same channel and other neurons. The energy function with regular terms added is defined as:

(2) $$e_{t}\left(w_{t},b_{t},y,x_{i}\right)=\frac{1}{M-1}\sum_{i=1}^{M-1}{\left(-1-\left(w_{t}x_{i}+b_{t}\right)\right)}^{2}+{\left(1-\left(w_{t}t+b_{t}\right)\right)}^{2}+\lambda w_{t}^{2}$$

There are M=H×W energy functions in the neural network. However, Equation (2) has an analytic solution:

(3) $$\begin{array}{l} w_{t}=-\frac{2\left(t-\mu_{t}\right)}{{\left(t-\mu_{t}\right)}^{2}+2\sigma_{t}^{2}+2\lambda }\\ b_{t}=-\frac{1}{2}\left(t+\mu_{t}\right)w_{t} \end{array}$$

where $u_{t}=\frac{1}{M-1}\sum_{i=1}^{M-1}x_{i},\sigma_{t}^{2}=\frac{1}{M-1}\sum_{i=1}^{M-1}{\left(x_{i}-u_{t}\right)}^{2}$. Therefore, the minimum energy can also be expressed as:

(4) $$e_{t}^{*}=\frac{4\left({\hat{\sigma }}^{2}+\lambda \right)}{{\left(t-\hat{\mu }\right)}^{2}+2{\hat{\sigma }}^{2}+2\lambda }$$

It can be concluded from Equation (4) that with the decrease in neuron energy, the difference between neurons and their peripheral neurons increases, and the importance increases accordingly. Therefore, the importance of neurons can be obtained by $1/e_{t}^{*}$. According to the means of attention mechanism, feature enhancement can be defined as:

(5) $$\tilde{X}=sigmoid\left(\frac{1}{E}\right)⊙X$$

There are some branches for the detection of small-scale objects in the latest YOLO series of algorithms. As the detection network is constructed for big coal blocks in the scraper conveyor, the existence of small- and medium-sized branches in the YOLO-BS network model has no practical value and will increase the number of parameters in the model. There are only two large-scale branches with feature scales of 26 × 26 and 13 × 13 in the YOLO-BS algorithm.

#### 4.1.4. Loss Function to Solve Sample Distribution Imbalance

In the normal production functioning of a scraper conveyor in a coal mine, the probability of big coal blocks is very low. Under normal circumstances, a scraper conveyor transports small coal blocks. This phenomenon will cause the detection dataset faced by the final algorithm to be dominated by negative samples of small coal. Therefore, in the dataset of this paper, the proportion of big coal block image data should be low, and there should be a large number of background images of small coal. Such a dataset composition is prone to data imbalance problems, which will affect the target segmentation of positive obstacles. With the aim to solve this problem, we proposed the use of focal loss [39] to optimize the cross-entropy loss function. Its mathematical expression is shown in Equation (6):

(6) $$\mathrm{Focal}\;\mathrm{loss}=\left\{ \begin{array}{l} -\alpha {\left(1-p\right)}^{\gamma }\log\left(p\right),\;\mathrm{if}\;\;y=1\\ -\left(1-\alpha \right)p^{\gamma }\log\left(1-p\right),\;\mathrm{if}\;y=0 \end{array}\right.$$

In Equation (6), y = 1 represents correct classification, y = 0 represents wrong classification, γ is used to adjust the weight between difficult and easy-to-classify samples, α is used to adjust the weight between positive and negative samples, and p represents the probability obtained by the model after classification. In this paper, we use the default weights of focal loss, namely α=0.25 and γ=2.

### 4.2. Calculation of the Abnormal Value of Coal Block in Scraper Conveyor

**Figure 5:**
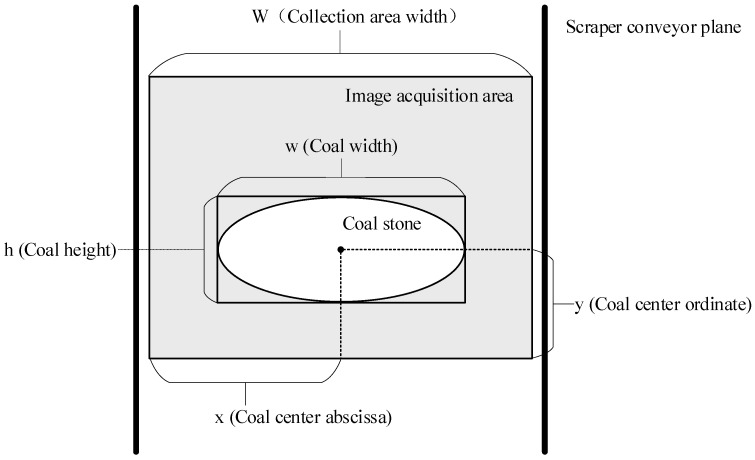
The position of coal on the scraper conveyor.

In the actual production of a mine, whether the coal flow of the scraper conveyor is congested not only depends on whether there are big coal blocks on the scraper conveyor, but also depends on the size, location, and quantity of the coal on the scraper conveyor. Coal blocks of the same size are likely to be located in the middle of the scraper conveyor without causing coal outlet congestion, but coal located on both sides of the scraper conveyor will cause coal outlet congestion. Using the YOLO-BS algorithm, the length, width, and center coordinates of the big coal blocks in the acquired image area can be detected, as shown in Figure 5. Through the analysis of the historical big coal block congestion phenomenon, in this paper, we define the congestion rate of the scraper conveyor:

(7) $$\mathrm{rate}=\left\{ \begin{array}{l} 0\;\;\;\;\;n=0\\ k\frac{w\times h}{\left|x-\frac{W}{2}\right|}\;\;\;\;\;\;\;n\leq n_{0}\\ 1{\;\;\;\;\;n>n}_{0} \end{array}\right.$$

In Equation (7), W represents the width of the camera acquisition area, n is the number of big coal blocks detected, x and y are the coordinates of the coal core, w and h represent the length and width of the coal body, respectively, and k and n_0_ are hyperparameters. When an abnormality of big coal blocks is detected in three consecutive frames of images, an abnormal alarm will be issued.

## 5. Monitoring and Analysis of Big Coal Blocks in Scraper Conveyor

### 5.1. Introduction to the Environment of Underground Mines

Daliuta Coal Mine is an extra-large, modern, high-yield, and efficient mine owned by the Shendong Coal Group, with an annual output of 20 million tons. It was the first underground mine built by the Shendong Coal Group. It is located in Shenmu County, Shaanxi Province, with an annual production capacity of 21.7 million tons. It is the only mine of Shendong Company listed as the first batch of intelligent demonstration mines by the state. The coal seam thickness of the working face in Daliuta Coal Mine is 4–6 m, and the working face width is 305.4 m. The longitudinal joints of the coal seam in this area are relatively developed. When pressure is applied to the working face, it easily breaks up and produces large lumps of coal; at the same time, due to the heavy mining operation, the shearer pulls quickly. After the coal machine is cut, the guard plate is not opened in time, and the coal wall cannot be supported in a timely and effective manner, causing large lumps of coal to be generated in the gang. The on-site scraper conveyor is the 3 × 1200 kW model of Zhangjiakou Coal Machinery Factory; the inner groove width is 1000 mm, and the speed of the scraper conveyor is 1.89 m/s.

### 5.2. Big Coal Block Monitoring System Equipment Installation and Data Collection

**Figure 6:**
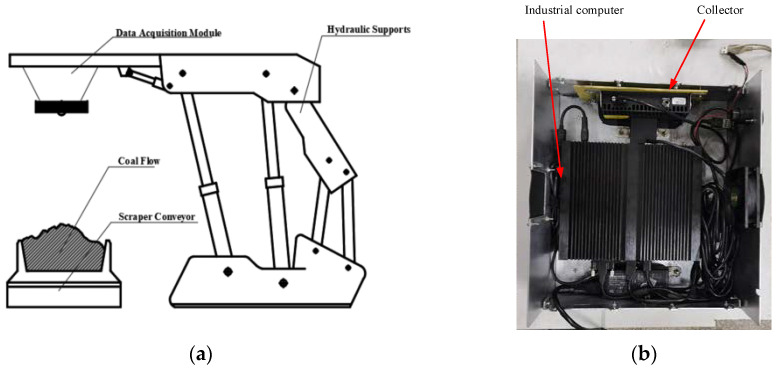
Data acquisition equipment: (**a**) equipment installation diagram; (**b**) data acquisition equipment.

The main data acquisition equipment used in this paper was an industrial computer and camera. The installation position of the camera acquisition equipment is shown in Figure 6a. Table 1 shows parameters of the camera used in this paper. Figure 6b shows the internal composition of the main information processing equipment used in this paper, which was an industrial computer and a power supply lamp. In addition, Table 2 also lists the model of the industrial computer and its equipment parameters.

Starting from 29 July 2021, in the normal production process, we filmed the coal flow at the head of the scraper conveyor using the camera for one week, and then we extracted 12,000 pictures of abnormal coal blocks. Each picture showed a different abnormal coal block scenario. The proportion of training set, verification set, and test set was 6:2:2; that is, 7200 pictures were used for the training set, 2400 pictures were used for the verification set, and 2400 pictures were used for the test set. Finally, we used the pictures obtained in the actual well as the dataset of this study, and we verified the correctness and practicality of the algorithm proposed in this paper.

### 5.3. Implementation Details

The computer configuration used in this experiment was Intel^®^ Core™ i9-10900K CPU, NVIDIA RTX 3090 (24G) GPU (Intel, Santa Clara, CA, USA), and the operating system was Windows 10 Professional Edition. The network model of this experiment was built based on the Pytorch 1.2 framework, and we used the transfer learning method. In the experiment, we used four indicators of precision, average precision (AP), and mAP as the standard for model accuracy evaluation:

(8) $$Pression=\frac{TP}{TP+FP}$$

(9) $$\mathrm{AP}=\frac{\sum_{1}^{n}\mathrm{Pression}\times \mathrm{Reacll}}{N}$$

(10) $$\mathrm{mAP}=\frac{\sum_{1}^{n}AP}{n}$$

In this formula, TP is the correctly detected target; FP is the background error detected as the target; FN is the undetected target; n is the category of target detection; N is the number of detected obstacles; AP measures the model accuracy of detection of negative obstacles; and mAP is the average of all AP and is used to measure the detection accuracy of the entire model.

### 5.4. Experiment

#### 5.4.1. Model Training

**Figure 7:**
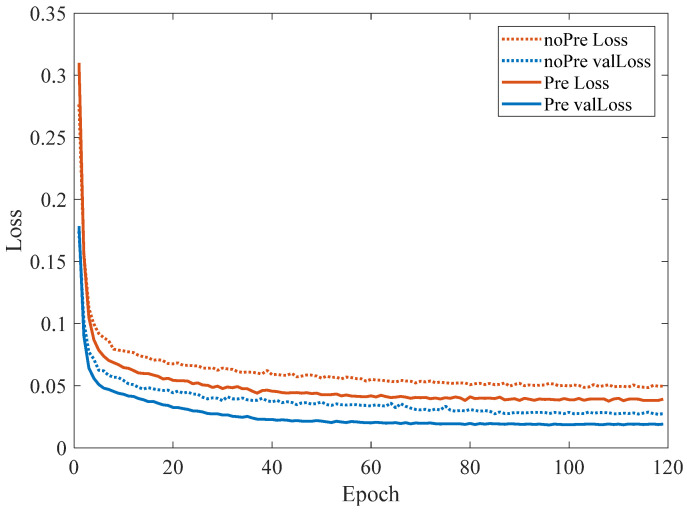
Big coal block dataset training loss curve.

**Figure 8:**
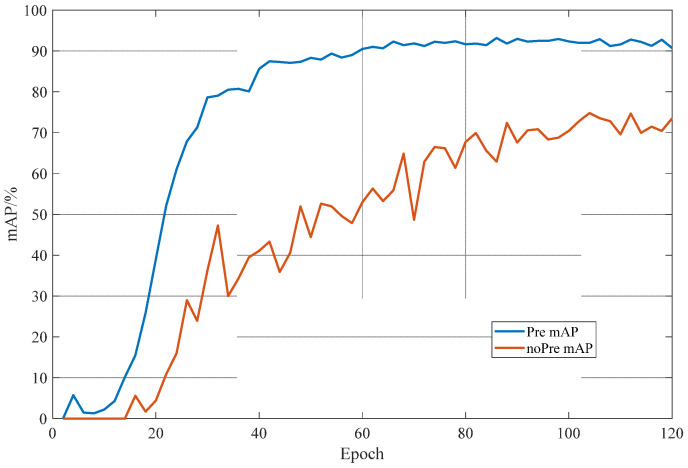
Big coal block dataset training mAP curve.

Transfer learning enables a network model to acquire a large number of common low-level image features, and at the same time, it can realize the reuse of low-level features and the fine-tuning of high-level rushing features in downstream tasks. To determine whether transfer learning is effective for the detection of big coal blocks, in this paper, we used the YOLO-BS model without transfer learning parameters and the YOLO-BS model with the VOC2007 + 2012 dataset as transfer learning parameters for large blocks. The coal body was detected and identified, and the results are shown in Figure 7. As can be seen from Figure 7, the use of transfer learning in the training set and validation set of big coal blocks can speed up the convergence of the YOLO-BS model and can stabilize the model loss to a value within a smaller range. Figure 8 shows the change in the mAP index of the model during the training process. It can be seen that the model using the transfer learning pre-training weight can make the mAP index of the model increase rapidly and stably compared with the model without the pre-training weight of migration learning.

#### 5.4.2. Model Test

To improve the feature information loss during the sampling process on the model, in this paper, we introduced the SimAM attention mechanism described in Section 4.1.3 into PANet. As shown in Table 3, we selected SE [40,41], CBAM [41,42], ECA [42,43], and the comparison of the calculation amount and parameter increase in the YOLO-BS model after the SimAM attention mechanism. FLOPs (floating point operations) refer to floating point operands, which can be understood as computational complexity and can be used to measure the complexity of algorithms/models. The smaller the FLOPs, the smaller the amount of calculation required for the model, and the faster it runs. It can be seen that the SimAM module can have better performance without increasing the number of parameters and FLOPs.

**Figure 9:**
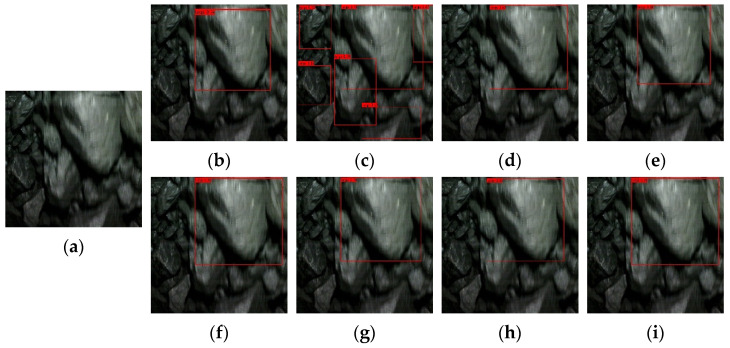
Results of big coal block detection via different networks—the first image. (**a**) origin; (**b**) SDD; (**c**) Faster-RCNN; (**d**) RetinaNet; (**e**) YOLOv3; (**f**) YOLOv4; (**g**) YOLOv5-L; (**h**) YOLOX-L; (**i**) YOLO-BS.

**Figure 10:**
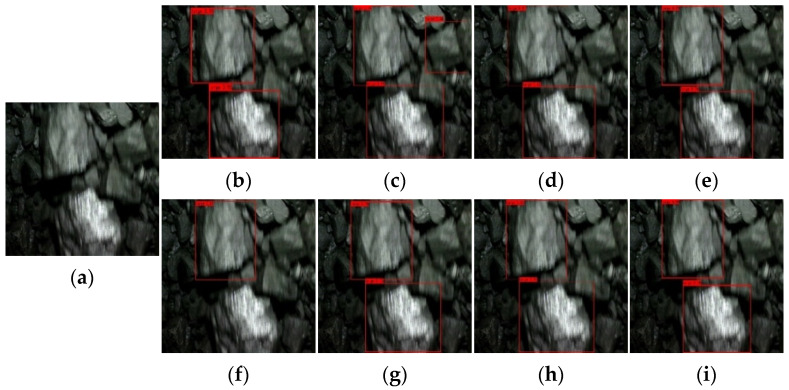
Results of big coal block detection via different networks—the second image. (**a**) origin; (**b**) SDD; (**c**) Faster-RCNN; (**d**) RetinaNet; (**e**) YOLOv3; (**f**) YOLOv4; (**g**) YOLOv5-L; (**h**) YOLOX-L; (**i**) YOLO-BS.

**Figure 11:**
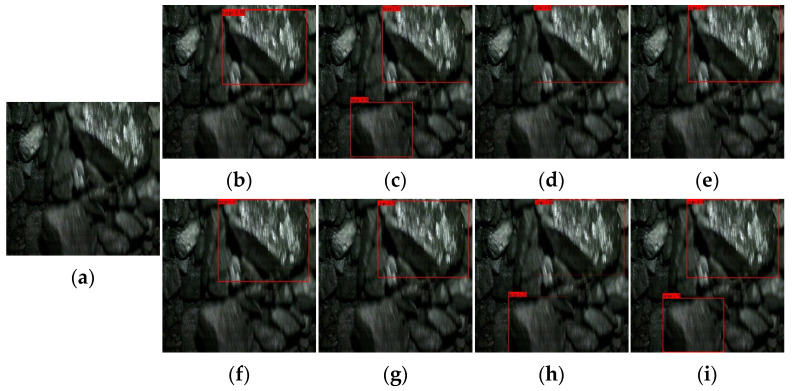
Results of big coal block detection via different networks—the third image. (**a**) origin; (**b**) SDD; (**c**) Faster-RCNN; (**d**) RetinaNet; (**e**) YOLOv3; (**f**) YOLOv4; (**g**) YOLOv5-L; (**h**) YOLOX-L; (**i**) YOLO-BS.

**Figure 12:**
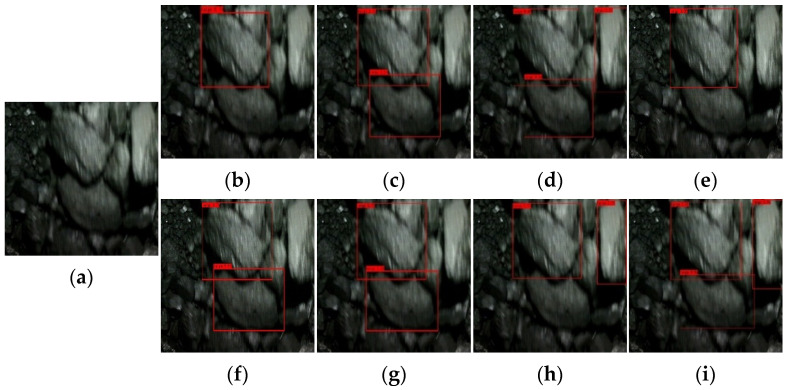
Results of big coal block detection via different networks—the fourth image. (**a**) origin; (**b**) SDD; (**c**) Faster-RCNN; (**d**) RetinaNet; (**e**) YOLOv3; (**f**) YOLOv4; (**g**) YOLOv5-L; (**h**) YOLOX-L; (**i**) YOLO-BS.

Figure 9, Figure 10, Figure 11 and Figure 12 show the comparative detection results of four types of images with the most camera records in actual production, including the current mainstream target detection networks SSD [43], Faster-RCNN [44], RetinaNet [39], YOLOv3 [45], YOLOv4 [35], YOLEOv5-L [46], YOOX-L [47], and YOLO-BS.

There is only one big coal in Figure 9, and this type of image should occur more frequently in the process of model detection. Most algorithms can accurately detect large lumps of coal, but Faster-RCNN identifies small lumps of coal that should not be identified. In Figure 10, there are two large coal stones that should have been identified. From the recognition results, Faster-RCNN incorrectly identified a small coal block that should not have been identified, while the YOLOv4 algorithm only identified one large coal. The coal block composition in Figure 11 is relatively complex, so it is difficult to identify. Only Faster-RCNN, YOLOX-L, and YOLO-BS can effectively identify large coal blocks. It can be seen from Figure 12 that the SSD algorithm and YOLOv3 algorithm have poor recognition effects, while RetinaNet and YOLO-BS accurately identify three large coal blocks.

By comparing the recognition effect of the YOLO-BS algorithm and seven mainstream comparison algorithm models in identifying four typical images, we can draw the conclusion that the YOLO-BS algorithm performs best in the detection of large coal and stone blocks. The Faster-RCNN algorithm can detect coal in an image effectively, but it provides no knowledge as to whether the coal identified is a large coal block. The recognition effect in the YOLOX-L and RetinaNet algorithms is better than in other algorithms, except YOLO-BS.

The YOLO-BS proposed in this paper was compared with the mainstream target detection networks, and the results are shown in Table 4. It can be seen from Table 4 that the proposed YOLO-BS model achieves the best mAP, precision, and Folps performance compared to other networks of the same type. The SDD model has the fewest parameters and can achieve the highest FPS performance of 112.96. However, due to the small size of the input image in its algorithm design, the low mAP and accuracy of the network cannot meet the detection requirements of this paper for large lumps of coal.

The superior performance of the YOLO BS network model is because it was actually customized for the identification of big coal blocks. According to the requirement of the global recognition ability of the bulk coal network model, a visual transformation (Vit) was added to the last layer of the model backbone network. From the results, the recognition accuracy and mAP of the model are greatly improved. Due to the characteristics of big coal blocks, only big-size targets needed to be identified, meaning the YOLO-BS framework was designed to delete the small-scale branches in the traditional YOLO algorithm. This operation greatly reduces the computational complexity and parameters of the model and improves the image processing speed of the model per second. In addition, the SimAM module and focal loss in the model also improve the recognition accuracy of the model to varying degrees.

#### 5.4.3. Analysis of Ablation Experiment

To more clearly analyze the impact of the improvement points in the proposed YOLO-BS algorithm on the algorithm, we conducted ablation experiments on the added ‘transform’, ‘branch deletion (Branch-d)’, and ‘SimAM’ modules.

From Table 5, we can see that both the ‘transform’ and ‘SimAM’ modules improve the algorithm in a positive way. However, the processing speed of the algorithm is low at this time. In order to improve the processing speed of the algorithm, we made a compromise in terms of Branch-d. From the final results, the overall effect of the proposed YOLO-BS algorithm met our design expectations.

### 5.5. Analysis of Monitoring Accuracy of Big Coal Blocks in Scraper Conveyor

**Figure 13:**
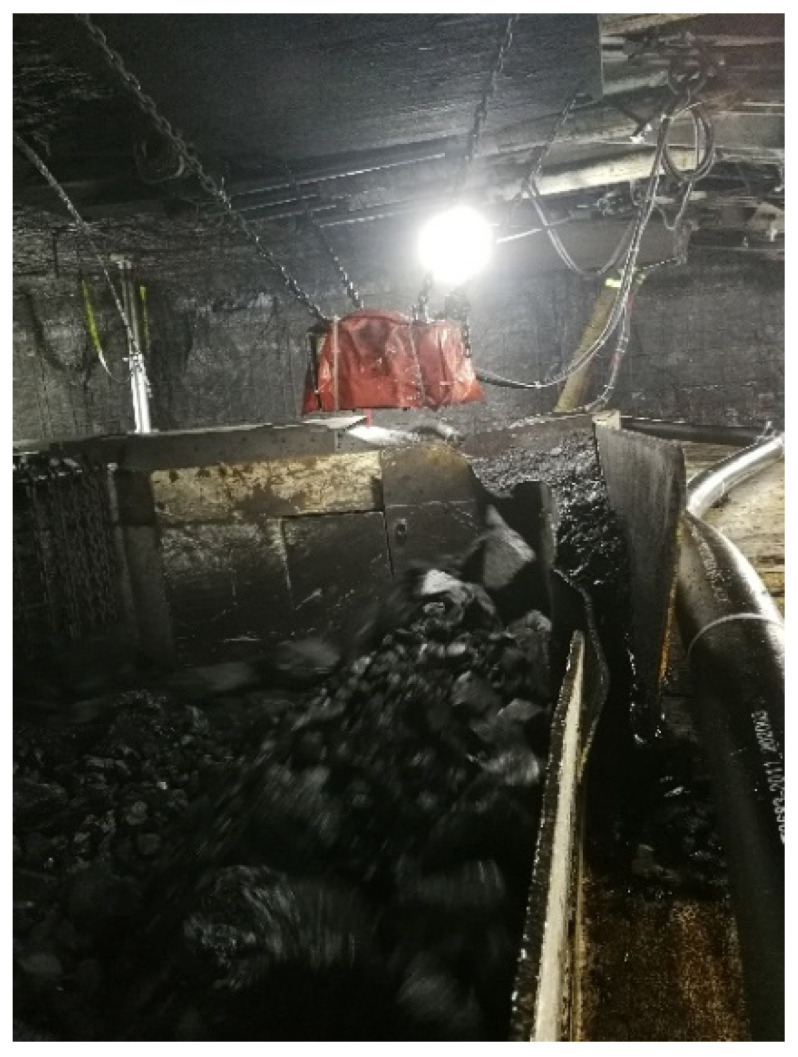
On-site work diagram of scraper conveyor.

To verify the effect of the algorithm on the monitoring of coal blocks in a real underground scraper conveyor, in this paper, we integrated the proposed YOLO-BS algorithm on the industrial computer to monitor the scraper conveyor coal block, as shown in Figure 13. The camera films video data, and the system converts the video data into image data and inputs it into the YOLO-BS algorithm for the detection and calculation of big coal blocks. Then, using the abnormal calculation method for big coal blocks proposed in this paper, the abnormal time period for big coal blocks on the scraper conveyor is calculated, and an alarm is sounded.

After a day of testing, only the YOLO-BS algorithm was used to judge whether coal outlet congestion would be caused by the size of the large coal block in the image, and the correct early warning rate could reach 91.3%. The YOLO-BS algorithm matches the blockage rate formula of the scraper conveyor proposed in Section 4.2 of this paper, and the accuracy of the large coal alarm is up to 93%.

### 5.6. Comparative Analysis of Advantages of Different Categories of Methods

To conduct a qualitative analysis regarding the method of processing large coal and stone blocks based on deep learning, we used our method and two other methods to solve large-coal-block-related problems, including the traditional image method (T-I method) and the lidar method. Table 6 shows our comparison results in terms of the four dimensions of economic cost, calculation power, detection speed, and accuracy.

In terms of economic cost, the purchase of lidar equipment and the subsequent processing of point cloud data are costly. Our method based on deep learning requires the purchase of better computer equipment. The equipment used in traditional image processing methods and the computer required in the processing algorithm are relatively basic, so their economic cost is not too high. Both our method and the lidar method require high calculation power. The T-I method is limited by its own algorithm, and its detection speed can basically meet the detection requirements. The detection effect of lidar is not ideal because of the complexity of data processing. Compared with the T-I method, the lidar method and the method in this paper have relatively complex and detailed processing processes, which mean they can more effectively obtain information regarding large lumps of coal from the original data, so the latter have higher detection accuracy.

## 6. Conclusions

With the aim of solving the problem of big coal blocks on scraper conveyors caused by the unfixed mining frequency of shearers, in this paper, we proposed a coal flow monitoring algorithm based on the YOLO-BS algorithm for use in scraper conveyors. The YOLO-BS algorithm and the same type of target detection algorithm show great advantages in the identification of big coal blocks, especially in terms of the mAP target of big coal blocks, as this reaches 96.86%. In the example application, the correct alarm rate of the monitoring system based on the YOLO-BS algorithm was as high as 93%, and the processing speed could reach 80 fps, which met the precision and real-time requirements for the intelligent monitoring of abnormally big coal blocks.

Due to the limitation of economic cost, the camera used in this paper had an insufficient shooting frame rate, and the lighting equipment was not installed properly, resulting in some blurred images being obtained, which affected the detection accuracy of the latter algorithm. Although we monitored the scraper conveyor coal block, the feedback to the shearer had a long delay, and the shearer was not adjusted in time when the monitoring equipment detected a big coal block.

To collect the best-quality scraper conveyor coal images, our team will use better camera equipment and more suitable lighting arrangements in the future. In response to the problem of untimely feedback from the shearer, our team plans to design a small coal block crusher to assist the big coal block monitoring system and solve this problem.

## Figures and Tables

**Table 1:** Camera parameters.

Parameter Type	Unit	Parameter
Image sensor	-	1/2.7 inch HM2131
Image pixel	-	2M 1080P
Image format	-	YUV/JPG
Camera lens	mm	F.3.0 f/no.24,000
Working current	mA	<200 mA
Sleep current	mA	<10 mA
Operating temperature	°C	−20~50

**Table 2:** Industrial computer parameters.

Parameter Type	Unit	Parameter
Model	-	MIC-7700
CPU	-	i7-6700T
Graphics card	-	Intel HD
System memory	GB	16 (DDR4 2400 MHz)
BIOS	-	AMI BIOS, ASPI supported
Power	V	DC 12 V
COM	-	6 × RS232

**Table 3:** Comparison of different attention mechanisms.

Attention	FLOPs	Param	mAP
Original	48,516,594,022	54,943,070.0	96.11
SENet	+239,616	+32,768	96.27
CBAM	+469,348	+65,634	96.41
ECA	+179,200	+5	96.31
SimAM	+0	+0	96.35

**Table 4:** Comparison of different network performances.

Model	Input Size	mAP/%	Precision/%	Prams/M	FLOPs/G	FPS
SSD	300 × 300	84.18	74.44	26.285	62.798	112.96
Faster-RCNN	600 × 600	86.04	46.54	137.099	370.406	21.77
RetinaNet	600 × 600	90.86	73.14	37.969	169.821	43.54
YOLOv3	416 × 416	85.74	82.70	61.949	66.096	65.79
YOLOv4	416 × 416	90.09	89.36	64.363	60.334	61.34
YOLOv5-L	640 × 640	90.52	83.95	47.057	115.603	72.12
YOLOX-L	416 × 416	89.03	83.13	54.209	65.762	64.45
YOLO-BS	416 × 416	96.86	96.10	57.303	49.315	80.68

**Table 5:** Comparative analysis of ablation.

Transform	Branch-d	SimAM	Focal Loss	mAP/%	Precision/%	Prams/M	Flops/G	FPS
--	--	--	--	90.09	89.36	64.363	60.334	61.34
√	--	--	--	95.66	93.18	59.112	58.559	63.73
√	√	--	--	94.13	91.86	54.943	48.517	79.97
√	√	√	--	96.35	94.35	57.303	49.315	80.68
√	√	√	√	96.86	96.10	57.303	49.315	80.68

**Table 6:** Performance comparison of different types of methods..

Contrast Item	T-I Method	Our Method	Lidar Method
Economic cost	low	middle	high
Calculation power	low	high	high
Detection speed	middle	high	middle
Accuracy	middle	high	high

## Data Availability

Not applicable.
